# Postabortion care service availability, readiness, and access in Burkina Faso: results from linked female-facility cross-sectional data

**DOI:** 10.1186/s12913-023-10538-z

**Published:** 2024-01-17

**Authors:** Yentéma Onadja, Rachidatou Compaoré, Danielle Belemsaga Yugbaré, Haley L. Thomas, Georges Guiella, Siaka Lougué, Henri Gautier Ouedraogo, Fiacre Bazie, Seni Kouanda, Caroline Moreau, Suzanne O. Bell

**Affiliations:** 1 Institut Supérieur des Sciences de la Population (ISSP), Université Joseph Ki-Zerbo, Ouagadougou, 03 BP 7118 Burkina Faso; 2 Institut de Recherche en Sciences de la Santé (IRSS), Ouagadougou, Burkina Faso; 3 Department of Population, Family and Reproductive Health, Johns Hopkins Bloomberg School of Public Health, Baltimore, MD USA; 4 Soins Primaires et Prévention, CESP Centre for Research in Epidemiology and Population Health, U1018, Inserm, Villejuif, F-94800 France

**Keywords:** Postabortion care, Abortion, Burkina Faso, Survey, Health facilities

## Abstract

**Background:**

Little is known about postabortion care (PAC) services in Burkina Faso, despite PAC’s importance as an essential and life-saving component of emergency obstetric care. This study aims to evaluate PAC service availability, readiness, and accessibility in Burkina Faso.

**Methods:**

Data for this study come from the Performance Monitoring for Action (PMA) Burkina Faso project and the Harmonized Health Facility Assessment (HHFA) conducted by the Institut de Recherche en Sciences de la Santé and the Ministry of Health. PMA data from a representative sample of women aged 15–49 (*n* = 6,385) were linked via GPS coordinates to HHFA facility data (*n* = 2,757), which included all public and private health facilities in Burkina Faso. We assessed readiness to provide basic and comprehensive PAC using the signal functions framework. We then calculated distance to facilities and examined percent within 5 kms of a facility with any PAC, basic PAC, and comprehensive PAC overall and by women’s background characteristics.

**Results:**

PAC services were available in 46.4% of health facilities nationwide; only 38.3% and 35.0% of eligible facilities had all basic and comprehensive PAC signal functions, respectively. Removal of retained products of conception was the most common missing signal function for both basic and comprehensive PAC, followed by provision of any contraception (basic) or any LARC (comprehensive). Nearly 85% of women lived within 5 km of a facility providing any PAC services, while 50.5% and 17.4% lived within 5 km of a facility providing all basic PAC and all comprehensive PAC signal functions, respectively. Women with more education, greater wealth, and those living in urban areas had greater odds of living within 5 km of a facility with offering PAC, basic PAC, or comprehensive PAC.

**Conclusions:**

Results indicate a need for increased PAC availability and readiness, prioritizing basic PAC services at the primary level—the main source of care for many women—which would reduce structural disparities in access. The current deficiencies in PAC signal a need for broader strengthening of the primary healthcare services in Burkina Faso to reduce the burden of unsafe abortion-related morbidity and mortality while improving maternal health outcomes more broadly.

## Background

Abortion is one of the safest procedures when performed according to recommended guidelines, resulting in less than 1 death per 100,000 abortions in high-resource settings [1–3]. However, unsafe abortion remains a leading cause of maternal mortality globally. Approximately 25 million unsafe abortions occur annually worldwide [4] which are responsible for an estimated 8–15% of all maternal deaths [5, 6]. The risk of severe morbidity or mortality associated with unsafe abortion varies widely across time and contexts. The case fatality associated with unsafe abortion is 220 deaths per 100,000 procedures in low-resource settings compared to 30 per 100,000 in high-resource settings [7], while many other women experience complications ranging in severity [8]. This more than seven-fold increase in case fatality in low-resource settings is in part due to differences in the unsafe means used to terminate an unwanted pregnancy but is also a result of the availability and accessibility of quality postabortion care (PAC) services to treat complications.

PAC is an essential and life-saving component of emergency obstetric care [1], which focuses on the treatment of complications due to abortion and prevention of similar conditions through postabortion contraceptive counseling and services [9, 10]. Yet we often know little about PAC availability and facility readiness to provide this service in low-resource settings [11]. The global community called for increased availability of PAC to treat complications arising from spontaneous and induced abortion - regardless of legality - as early as 1994 at the International Conference on Population and Development in Cairo [12]. However, several decades later the provision of PAC services remains vastly inadequate [13–17].

Beyond service availability, several studies have sought to evaluate the readiness of facilities to provide *quality* PAC [13, 14, 17, 18] by examining a facility’s ability to provide all components of the service, an essential precursor to achieving quality of care [19]. These components typically include both structural and process elements, referred to as signal functions. In relation to safe abortion care (SAC), signal functions comprise curative and preventative elements, including safe-induced abortion care for all legal indications, treatment of complications, and provision of postabortion contraception [18]. Basic SAC is defined by the ability to provide abortion and PAC through 12 weeks gestation in conjunction with postabortion contraception, while comprehensive SAC requires the capacity to provide these services beyond 12 weeks, treat more serious complications requiring blood transfusion or abdominal surgery, and to offer long-acting reversible contraceptive (LARC) methods (intrauterine devices (IUDs) or implants). Campbell and colleagues later adapted these criteria for PAC specifically [20]. Recent studies of PAC in sub-Saharan Africa find consistently low levels of service readiness, from less than 10% in Namibia, Rwanda, and Uganda to 33% and 37% in Nigeria and Cote d’Ivoire, respectively [13, 14, 16]. The assessment of basic and comprehensive PAC signal functions has important programmatic utility as a component of quality obstetric care.

In Burkina Faso, approximately 113,000 induced abortions occur each year and 90% are estimated to be unsafe [21]. Many women with unsafe abortions require treatment for incomplete abortions or complications that arise, and thus, need access to quality PAC [8]. A study of PAC conducted in 2018/19 in Burkina Faso found that 65% of primary-level facilities in the study sample had all basic PAC signal functions (excluding having a vehicle for transport), while 33% of referral facilities had all comprehensive signal functions (again, excluding transport capability) [15]. While this study provided a good overview of PAC availability and readiness, it relied on a sample of only 414 public facilities with no ability to examine PAC accessibility for women of reproductive age. Another cross-sectional study conducted across 11 countries in different sub-Saharan African regions in 2017/18 found high levels of capability for the provision of standard comprehensive PAC signal functions in West African countries, including Burkina Faso [17]. However, this study relied on pooled data from 210 public health facilities across these countries, which may mask important differences between countries and provides no insights regarding the private for-profit sector. To address these limitations, the current study relies on data from a census of public and private health facilities and links facility data to a contemporaneous nationally representative sample of reproductive aged women in Burkina Faso, allowing us to assess disparities in access to quality PAC services.

The objectives of this study were to evaluate PAC availability and readiness in Burkina Faso using the signal functions framework and to evaluate the accessibility of PAC services by women’s background characteristics. We also sought to determine the extent to which administrative regions achieved the World Health Organization’s (WHO) recommended service-to-population ratio among facilities offering this obstetric service. This health systems examination of PAC services will provide specific guidance on areas for improvement that can help address abortion-related morbidity and mortality in Burkina Faso.

## Methods

### Data

Data for this study come from Performance Monitoring for Action (PMA) Burkina Faso project [22] and the Harmonized Health Facility Assessment (HHFA) conducted by the Institut de Recherche en Sciences de la Santé (IRSS) and the Ministry of Health with support from the WHO. PMA conducts rapid turnaround nationally representative family planning surveys annually [23]. For this study, we used the PMA Burkina Faso Phase 2 female data collected from December 2020 through March 2021. PMA uses a multi-stage cluster sampling design with probability proportional to size sampling of urban/rural stratified enumeration areas (EAs), which are geographic units defined by the central statistics office that consist of approximately 200 households [23]. Resident interviewers generated a master sampling frame of all households in each EA then randomly selected 35 households within each area; all women of reproductive age (15 to 49 years old) in selected households were invited to participate. Interviewers conducted surveys face-to-face in French or a local language and entered responses via smartphone. The female response rate was 93.4%. The female survey included questions on women’s socioeconomic characteristics, as well as their reproductive history, knowledge of and attitudes towards family planning, and contraceptive experiences. The Phase 2 female survey also contained an abortion module that included questions on abortion knowledge and attitudes and respondent’s and their closest female friend’s experiences with abortion. In accordance with the relevant guidelines and regulations, women provided verbal informed consent prior to participating. This study was approved by the Johns Hopkins University Bloomberg School of Public Health institutional review board and the Comité d’Ethique pour la recherche en santé/ Ministère de la Santé et Ministère de l’Enseignement Supérieur, de la Recherche Scientifique et de l’Innovation in Burkina Faso.

The HHFA survey consisted of a census of all public and private health facilities in Burkina Faso. Data collection for the HHFA survey took place from November 23, 2020 to January 10, 2021 in the 13 administrative regions of Burkina Faso. Researchers identified 3,056 health facilities, of which 2,757 completed surveys (90.2% response rate). The remaining 299 health facilities could not be surveyed because they were non-functional, closed, or inaccessible for security reasons. The IRSS team adapted the WHO-developed HHFA questionnaires to the country context and the needs of the study. These questionnaires consisted of modules addressing several health topics, including structural features of the facility, provider information, maternal and child health, family planning, legal SAC, PAC services, communicable disease, and quality of care.

For the current study, we focused on PAC service-specific readiness by measuring the signal functions needed to provide basic and comprehensive PAC (Table 1). Each signal function was a binary variable, which we coded as available if the necessary equipment or commodity was available (and functioning in the case of equipment) on the day of the survey, or if an appropriately trained provider worked at the facility in the case of the removal of retained products of conception signal function. For removal of retained products of conception, we indicated this service was available for gestations 12 weeks or less if the facility reported providing provision of treatment for incomplete abortion, manual vacuum aspiration (MVA), or electric vacuum aspiration (EVA); for pregnancies of higher gestations, we used information on whether the facility provided dilation and evacuation (D&E, which was asked in the context of miscarriage management) or provision of dilation and curettage (D&C). Although the WHO no longer recommends the provision of D&C, we included it as it is very commonly used in low-resource settings and still has a strong safety profile for treating abortion complications. We defined “any PAC” as removal of products of conception.

**Table 1:** Basic and comprehensive PAC signal functions criteria

**Basic**			
	≤ 12 weeks removal of retained products		
	Antibiotics		
	Uterotonics		
	Intravaneous replacement fluids		
	Any contraception		
**Comprehensive**			
	All basic signal functions		
	> 12 weeks removal of retained products		
	Blood transfusion		
	Laparotomy		
	24/7 PAC services available		
	LARC		

We linked female and facility data using geospatial information. For each woman and facility, interviewers captured the Global Positioning System (GPS) point at the time of the interview. We used Euclidean distance to link each woman to each facility sampled and then determined the closest surveyed facility that provided any PAC, had all basic PAC signal functions, and had all comprehensive PAC signal functions.

### Analysis

To synthesize information on facility type, we grouped facilities by level. Tertiary facilities consisted of university, regional, and private hospitals; secondary facilities included district hospitals and private health facilities; primary facilities included doctor’s offices, medical centers, centres de santé et de promotion sociale (CSPS), maternity clinics, and isolated maternity facilities. We determined these facility groupings based on our knowledge of the local healthcare system and the Ministry of Health’s description of the functions that specific facility types are expected to provide. In all, 2,583 primary health facilities, 147 district hospitals and private health facilities, and 27 university, regional and private hospitals should have the capacity to provide basic PAC, while only the district hospitals and private health facilities (*n* = 147), and the university, regional and private hospitals (*n* = 27) are expected to have the capacity to provide comprehensive PAC.

We conducted analyses to evaluate PAC availability, readiness, and accessibility; basic PAC was evaluated among all facilities and comprehensive PAC was evaluated among referral facilities only. We used facility reports of PAC provision to assess PAC availability overall and by facility characteristics, including type, managing authority, and administrative regions. Among facilities offering PAC, we examined the availability of each basic and comprehensive signal function overall and by facility characteristics. Next, we determined whether each facility had all requisite signal functions for basic and comprehensive PAC and determined the percent of all facilities with all basic and comprehensive criteria, overall and by facility characteristics. For the female-facility linked analyses, we estimated the percent of women in Burkina Faso who live within 5 km (km) of a facility offering any PAC, a facility with all basic PAC signal functions, and a facility with all comprehensive PAC signal functions; we then examined PAC accessibility by women’s age (15–19, 20–29, 30–39, 40–49), level of education (none, primary, secondary, tertiary), household wealth (a tertile (poorest, middle, wealthiest) based on principal components analysis using information on household assets, materials, water, and sanitation residence (urban, rural) and region [21]. We then conducted a multivariable logistic regression to determine the independent odds of living within 5 km of a facility offering PAC services by women’s characteristics. Lastly, we used national and regional population data from the 2019 fifth general population and housing census of Burkina Faso [24] to determine the coverage of PAC in Burkina Faso in relation to the WHO recommendation of 5 facilities per 500,000 individuals for basic PAC and 1 facility per 500,000 individuals for comprehensive PAC.

All analyses were performed using Stata version 15.1 [25]. For the female-facility linked analyses we applied survey weights to account for the complex sampling design and calculated robust standard errors to account for clustering.

## Results

### PAC service availability

Results indicate that PAC services were available in 46.4% of health facilities nationwide, with substantial variability by facility characteristics and location (Table 2). PAC services were offered in 95.8% of tertiary facilities, 87.6% of secondary facilities, and 44.0% of primary health facilities. Many more private facilities provided PAC (54.5%) compared to public facilities (45.6%). Examining availability by facility location showed that PAC services were available in nearly seven in ten facilities located in urban areas (69.9%) but only 40.7% of facilities in rural areas. Analysis by region revealed large differences in the availability of PAC across the country. Sahel region (86.2%) had the highest percentage of health facilities with available PAC services while Plateau Central region was the region with the fewest health facilities providing PAC (28.3%) (Table 2). Between 35% and 70% of facilities in the other 11 regions offered PAC.

**Table 2:** Percentage of facilities in Burkina Faso offering PAC services, by facility characteristics, HHFA survey, 2020

		Facilities		
		%	N	Chi2
**Facility Type**				**89.23**
	Tertiary facilities	95.8	24	*p* < 0,001
	Secondary facilities	87.6	89	
	Primary facilities	44.0	2068	
**Managing Authority**				**5,52**
	Public	45.6	1990	*p* = 0.0188
	Private	54.5	191	
**Location area**				**117.50**
	Rural	40.7	1756	*p* < 0,001
	Urban	69.9	425	
**Region**				**130.14**
	Boucle du Mouhoun	51.1	231	*p* < 0,001
	Cascades	62.3	106	
	Centre	58.5	205	
	Centre-Est	47.6	185	
	Centre-Ouest	37.6	250	
	Centre-Nord	41.0	156	
	Centre-Sud	36.0	136	
	Est	67.0	112	
	Hauts-Bassins	44.8	230	
	Nord	35.0	203	
	Plateau Central	28.3	173	
	Sahel	86.2	58	
	Sud-Ouest	47.1	136	
**Total**		**46.4**	**2181**	

### PAC signal functions and service readiness

Table 3 presents the availability of individual PAC signal functions across facility types among facilities that reported offering PAC. Removal of retained products of conception was the most common missing basic signal function, with only 57.0% of all facilities reporting providing this service; the percentage with this function was lowest among primary facilities (54.8%), higher for secondary facilities (89.8%), and highest for tertiary facilities (100.0%). The availability of any contraceptive method was the next least available signal function at 85.9%, with secondary (58.2%) and tertiary facilities (75.0%) less likely to provide the service than primary facilities (87.4%). More than nine out of ten (96.1%) facilities had uterotonics and more than 85% had intravenous replacement fluids (88.7%) and antibiotics (86.5%); however, these three commodities were least available in secondary facilities. The ability to provide removal of retained products of conception for pregnancies beyond 12 weeks gestations (56.6%) and 24/7 PAC service (68.0%) were among the most commonly missing comprehensive signal functions among secondary and tertiary facilities expected to provide comprehensive PAC. Approximately 85% of higher-level facilities were able to provide blood transfusion (83.7%), perform laparotomy (85.6%), while only 58.2% offered LARCs. For all comprehensive signal functions, tertiary facilities were more likely to meet the criteria than secondary facilities.

**Table 3:** Percentage of all facilities that have specific basic and comprehensive PAC signal functions, HHFA Survey 2020

		Tertiary facilities (*n* = 24)	Secondary facilities (*n* = 98)	Primary facilities (*n* = 1934)	Total (*n* = 2056)
		%	%	%	%
Basic					
	≤ 12 weeks removal of retained products	**100.0**	**89.8**	**54.8**	57.0
	Antibiotics	**83.3**	**70.4**	**87.4**	86.5
	Uterotonics	**95.8**	**82.7**	**96.8**	96.1
	Intravaneous replacement fluids	95.8	82.7	88.9	88.7
	Any contraception	**75.0**	**58.2**	**87.4**	85.9
Comprehensive (basic +)					
	> 12 weeks removal of retained products	70.8	53.1	−	56.6
	Blood transfusion	**100.0**	**79.0**	−	83.7
	Laparotomy	91.3	84.0	−	85.6
	24/7 PAC services available	**87.5**	**63.3**	−	68.0
	LARC	66.7	56.1	−	58.2

^a^ Bold values indicate statistically significant difference at the *p* < 0.05 level based on Chi2 test

Results presented in Table 4 illustrate the percentage of facilities with all basic and comprehensive PAC signal functions. Although all health facilities should have the capacity to provide at least basic PAC services, only 38.3% had all basic PAC signal functions. Tertiary facilities (62.5%) were most likely to have these readiness criteria while secondary (34.7%) and primary facilities (38.2%) had similarly low percentages of facilities with all criteria. Public facilities (41.9%) were more than seven times more likely to have all basic PAC signal functions compared to private facilities (5.5%). While there was no difference in relation to urban versus rural facility location, there was marked variability by region of the country, with the highest proportion of facilities that had all basic PAC signal functions in Cascades (69.5%) and the lowest in Centre (22.5%).

**Table 4:** Percentage of facilities with all basic and all comprehensive PAC signal functions, by facility characteristics, HHFA Survey 2020

	Basic		Comprehensive	
	%	N	%	N
Facility Type				
Tertiary facilities	**62.5**	24	**54.2**	24
Secondary facilities	**34.7**	98	**30.6**	98
Primary facilities	**38.2**	1934	−	−
Managing Authority				
Public	**41.9**	1854	**66.1**	59
Private	**5.5**	202	**6.4**	63
Location Area				
Rural	38.6	1632	40.0	5
Urban	37.3	424	35.0	117
Region				
Boucle du Mouhoun	**45.9**	218	**71.4**	7
Cascades	**69.5**	105	**25.0**	4
Centre	**22.5**	187	**21.9**	32
Centre-Est	**45.7**	175	**27.3**	11
Centre-Ouest	**43.4**	219	**20.0**	10
Centre-Nord	**31.3**	134	**25.0**	4
Centre-Sud	**32.1**	134	**100.0**	4
Est	**59.3**	113	**50.0**	6
Hauts-Bassins	**35.2**	230	**22.7**	22
Nord	**22.9**	188	**100.0**	5
Plateau Central	**25.3**	166	**40.0**	5
Sahel	**58.9**	56	**40.0**	5
Sud-Ouest	**35.9**	131	**42.9**	7
Total	38.3	2056	35.3	122

^a^ Bold values indicate statistically significant difference at the *p* < 0.05 level based on Chi2 test

Among all higher-level facilities with the ability to provide comprehensive PAC services, only 35% had all comprehensive PAC signal functions (Table 4). The percent of facilities with comprehensive PAC signal functions varied by facility type, with only 30.6% of the secondary facilities and 54.2% of the tertiary-level facilities fulfilling these readiness criteria (Table 4). Similar to basic PAC readiness, public facilities (66.1%) were much more likely than private facilities to have all signal functions, there was substantial variability by region, and there was little difference in readiness between rural and urban facilities.

### Population coverage of PAC services

Based on the 2019 total Burkina Faso population figure of 20,505,155 [24], there are an estimated 19 health facilities with all basic PAC services per 500,000 inhabitants, and 1.05 facilities with all comprehensive PAC per 500,000 inhabitants (Table 5). This level of coverage is beyond the WHO recommendations of 5 facilities providing basic PAC and 1 facility providing comprehensive PAC per 500,000 population, respectively. Thus, the Burkinabè health system had 384% of the recommended number of facilities with the capacity to provide quality basic PAC services, and 105% of the recommended number of facilities with the capability to provide quality comprehensive PAC (Table 5). The percentage of facilities that met recommended levels varied widely by region, with the highest coverage level of basic PAC in the Cascades region at 898% and the lowest in the Centre region at 139%, though this is still above the recommended level (Table 5). The level of comprehensive PAC coverage was at or above recommended levels in six regions, ranging from 102% in Plateau Central to 254% in Centre-Sud. However, it was below 1 per 500,000 population in the other seven regions, ranging from 27% in the Centre-Nord region to 95% in the Centre-Est region.

**Table 5:** Recommended and actual numbers of health facilities with all basic and all comprehensive PAC signal functions, nationally and by region, HHFA Survey 2020

	Basic PAC			Comprehensive PAC		
	Recommended number	Actual number	%	Recommended number	Actual number	%
**National** **Region**	205	787	384	41	43	105
Boucle du Mouhoun	19	100	526	4	5	131
Cascades	8	73	898	2	1	62
Centre	30	42	139	6	7	116
Centre-Est	16	80	506	3	3	95
Centre-Ouest	17	95	573	3	2	60
Centre-Nord	19	42	224	4	1	27
Centre-Sud	8	43	550	2	4	254
Est	19	67	345	4	3	77
Hauts-Bassins	22	81	361	4	5	111
Nord	17	43	250	3	5	145
Plateau Central	10	42	429	2	2	102
Sahel	11	33	300	2	2	91
Sud-Ouest	9	47	537	9	3	34

### Access to PAC services among women of reproductive age

About 6 out of 10 women of reproductive age in Burkina Faso (56.1%) lived within 5 km of a facility providing any PAC services, while 50.5% lived less than 5 km from a facility with all basic PAC signal functions, and only 17.4% lived within 5 km of a facility with all comprehensive PAC signal functions (Table 6). We observed significant disparities in geographic access to these services. Women with no education (43.7%) were less likely to live near a facility with all basic PAC signal functions compared to those with higher education (94.7%). Women who lived in urban areas (95.3%) had greater geographic access to facilities with all basic PAC signal functions compared to those who lived in rural areas (37.7%). The poorest women (26.9%) were also significantly less likely to live within 5 km from a facility with all basic PAC signal functions compared to the wealthiest women (78.4%) (Table 6). Similar patterns were observed for access to facilities with all comprehensive signal functions; however, the percentage of women living within 5 km of facilities that met the comprehensive criteria was lower, regardless of the characteristics examined (Table 6).

**Table 6:** Percent of women living within 5 km of a facility providing any PAC or all basic or comprehensive PAC signal functions, by background characteristics, HHFA and PMA Surveys (*N* = 6,385)

		N	Any PAC	Basic PAC	Comprehensive PAC
Age (years)					
	15–19	1349	57.3	50.0	**18.3**
	20–29	2232	56.8	52.3	**19.4**
	30–39	1749	53.5	48.1	**16.4**
	40–49	1055	57.3	51.5	**14.1**
Education					
	Never	2681	**47.6**	**43.7**	**7.8**
	Primary	1298	**61.1**	**54.0**	**20.8**
	Secondary	2120	**70.4**	**61.4**	**34.1**
	Higher	284	**96.6**	**94.7**	**81.9**
Residence					
	Rural	2612	**43.7**	**37.7**	**0.8**
	Urban	3773	**99.1**	**95.3**	**75.4**
Wealth tertile					
	Poorest	1183	**29.3**	**26.9**	**0.7**
	Middle	1315	**50.5**	**46.0**	**3.2**
	Wealthiest	3887	**88.1**	**78.4**	**47.4**
Region					
	Centre	1606	**95.9**	**88.6**	**66.5**
	Hauts-Bassins	1582	**58.8**	**58.3**	**41.0**
	Other regions	3197	**50.0**	**44.0**	**7.2**
Total		6385	56.1	50.5	17.4

^a^ Bold values indicate statistically significant difference at the *p* < 0.05 level based on design-based F test

Results of multivariable logistic regression are shown in Table 7. Wealthier women were more likely to live near facilities with any PAC and those with all basic or comprehensive PAC signal functions, with aORs ranging from 1.72 to 2.45 for the middle wealth tertile and 3.62–6.81 for the wealthiest tertile when compared to the poorest women. Lastly, urban residence was positively associated with greater likelihood of living within 5 km from facilities that met each of the PAC criteria, with aORs ranging from 17.34 for a facility offering any PAC to 138.33 for a facility with all comprehensive PAC criteria, though confidence intervals were very wide.

**Table 7:** Adjusted odds ratio of living within 5 km of facility providing any PAC, or all basic or comprehensive PAC signal functions among reproductive aged women, HHFA and PMA Surveys (*N* = 6,385)

		Any PAC	Basic PAC	Comprehensive PAC
		aOR (95% CI)	aOR (95% CI)	aOR (95% CI)
Age (years)				
	15–19	(ref)	(ref)	(ref)
	20–29	0.91 (0.65–1.28)	1.00 (0.70–1.43)	0.93 (0.69–1.25)
	30–39	1.01 (0.64–1.60)	0.97 (0.61–1.53)	1.00 (0.82–1.22)
	40–49	1.44 (0.95–2.19)	1.28 (0.85–1.91)	1.05 (0.78–1.39)
Education				
	Never	(ref)	(ref)	(ref)
	Primary	1.16 (0.81–1.67)	0.95 (0.67–1.33)	0.98 (0.76–1.26)
	Secondary or higher	1.12 (0.65–1.93)	0.79 (0.48–1.30)	1.08 (0.78–1.50)
Residence				
	Rural	(ref)	(ref)	(ref)
	Urban	60.24 (16.26-223.14)	22.43 (5.34–94.19)	138.33 (9.74-1964.49)
Wealth tertile				
	Poorest	(ref)	(ref)	(ref)
	Middle	2.47 (1.56–3.89)	2.28 (1.37–3.80)	2.45 (1.19–5.05)
	Wealthiest	7.18 (2.85–18.08)	3.62 (1.32–9.96)	6.48 (1.73–24.32)
Region^a^				
	Centre	(ref)	(ref)	(ref)
	Hauts-Bassins	0.16 (0.02–1.05)	0.53 (0.11–2.64)	1.78 (0.31–10.35)
	Other regions	0.54 (0.08–3.40)	0.96 (0.17–5.58)	0.63 (0.14–2.81)

## Discussion

While PAC is widely available across the Burkina Faso health system, many facilities lack essential components needed to provide quality basic or comprehensive PAC, and there is disparate access to these life-saving services. Three-quarters of all facilities offered PAC, but less than four in ten of these facilities had all basic PAC signal functions; a similar proportion of secondary and tertiary facilities that should be able to provide comprehensive PAC had all the necessary components. Our comprehensive PAC readiness findings were consistent with a recent smaller study of PAC signal functions in Burkina Faso, but we observed much lower levels of readiness among primary facilities [15]. These findings are also broadly consistent with results from other sub-Saharan African countries regarding the presence of substantial gaps in basic and comprehensive PAC signal functions [13, 14, 16, 17]. The ability to remove retained products of conception was the signal function most often missing in our study, particularly at primary facilities. This is an essential aspect of PAC– and emergency obstetric care more broadly– needed to treat incomplete abortions and reduce the burden of unsafe abortion related negative sequelae [1]. A recent study of PAC in Burkina Faso showed that surgical PAC services for first- and second-trimester pregnancies were mostly hampered by a lack of trained providers and equipment [26]. In Burkina Faso, training in comprehensive PAC is generally provided by non-governmental organizations (NGOs), and the health workers to be trained are thus selected according to the criteria and programmatic priorities of these NGOs [26]. This results in a concentration of trained health workers in the regions where these NGOs implement their program activities.

For women who are experiencing abortion-related complications that require emergency facility-based treatment, they will likely need to travel beyond their nearest facility to access PAC, particularly for more severe complications. While primary facilities are often the closest facility and the first point of care in a medical emergency, we found only 38% of primary facilities had all basic PAC signal functions. Furthermore, our results highlight disparities in geographic proximity to PAC, particularly to a facility with the readiness to provide quality basic or comprehensive PAC; women with fewer financial resources, women residing in rural areas, and women with less education were least likely to live near a facility offering any PAC, and even less likely to live near a facility with all basic or comprehensive PAC signal functions. Evidence suggests rural women and women with the least education are most likely to have had an unsafe abortion in Burkina Faso, thus the women who are furthest from these services are most likely to require them [21]. Compounding typical delays in accessing care [27], the further distance these women must travel to access emergency obstetric care likely contributes to the greater documented burden of unsafe abortion-related morbidity and mortality that poor, rural women experience [28].

Postabortion contraceptive counseling and provision are part of the constellation of PAC services that aid in preventing future unintended pregnancies. However, 15% of all facilities were not providing any contraception, while 25% and 42% of tertiary and secondary facilities, respectively, were not doing so. These results are similar to those observed in other sub-Saharan Africa settings [13, 20]. Health workers often refer women to primary-level care for contraceptive counseling and methods, but this is a missed opportunity to meet contraceptive demand for women who are clearly fertile and do not wish to be pregnant. Research suggests providing family planning counseling and methods at the same time as PAC is best and leads to greater uptake, protecting them from subsequent unintended pregnancies [9, 29, 30]. Other work exploring receipt of PAC among women at facilities across 11 countries in Africa found 62% of women having at least one negative experience of care, which included things like not being able to ask questions during the treatment (34%), not feeling their preferences were followed during receipt of care (18%), not receiving pain medication (13%), and dissatisfaction with privacy and wait time (15% and 22%, respectively) [31]. These experiences– which were more likely among young women, unmarried women, and those with less education or wealth– offer a potential explanation for why many delay or avoid facility-based PAC even when they are concerned about complications [31]. Future work examining patient-centered measures of PAC is needed to systematically include this aspect of quality in subsequent research and monitoring of these services.

This study has several strengths. It provides a comprehensive assessment of PAC availability and readiness in Burkina Faso, relying on data from a census of all public and private health facilities in the country. Results reveal opportunities for health system strengthening at each level of the healthcare system as well as regions that require improvements in PAC services, including a need for greater coverage of comprehensive PAC in the regions that have not reached WHO standards. We were also able to leverage the contemporaneously collected facility and nationally representative female data to geographically link these data, enabling a unique analysis of access to services for reproductive aged women.

However, this study is not without limitations. While we sought to evaluate facility readiness to provide quality PAC, the presence of these signal functions does not ensure that women will receive quality care that is respectful and adheres to medical guidelines. Evidence suggests stigma and concerns about treatment from healthcare providers prevent some women from obtaining safe abortion or PAC services [32]. We do not have data on provider PAC training, nor do we observe the provision of these services to determine providers’ technical competence. We were also unable to directly measure PAC outcomes. In addition, our geographic indicator of accessibility using Bell et al.’s criteria of a 5 km distance from a health facility doesn’t capture other dimensions of accessibility, including economic and cultural conditions.

Despite these limitations, results from our study provide actionable information to improve PAC services in Burkina Faso. Efforts to increase PAC availability and readiness should prioritize basic PAC services at the primary level, the main source of care for many women. This would reduce structural disparities in access and reduce delays in receiving PAC while increasing the likelihood that women receive timely and quality PAC for treatment of abortion complications. The current deficiencies in PAC signal a need for broader strengthening of the primary healthcare services in Burkina Faso. Ensuring adequate provider training and the stock of essential medicines and devices will help reduce the burden of unsafe abortion-related morbidity and mortality while improving maternal health outcomes more broadly. Additional work must address the social aspects of unsafe abortion and PAC to increase women’s willingness to use these services [32].

## Data Availability

PMA data are publicly available and can be accessed by submitting a request through the PMA website: https://datalab.pmadata.org/. The authors used the Burkina Faso Phase 2 household/female datasets for this analysis (10.34976/28dw-kk71). HHFA data is available upon request from Assane Ouangaré (ouangarea@gmail.com) at the Burkina Faso Ministry of Health and Public Hygiene or Séni Kouanda (senikouanda@gmail.com) at the Institut de Recherche en Sciences de la Santé (IRSS).

## Abbreviations

PAC: Postabortion care
SAC: Safe abortion care
LARC: Long-active reversible contraceptive
IUD: Intrauterine device
WHO: World Health Organization
PMA: Performance Monitoring for Action
HHFA: Harmonized Health Facility Assessment
IRSS: Institut de Recherche en Sciences de la Santé
EAs: Enumeration Areas
MVA: Manual vacuum aspiration
EVA: Electric vacuum aspiration
D&E: Dilation and evacuation
D&C: Dilation and curettage
GPS: Global positioning system
km: kilometers
aOR: Adjusted odds ratio
NGO: Non-governmental organization
